# Liquid Biopsy in Thyroid Cancer: New Insight

**Published:** 2018-07-01

**Authors:** Fatemeh Khatami, Seyed Mohammad Tavangar

**Affiliations:** 1 Chronic Diseases Research Center, Endocrinology and Metabolism Population Sciences Institute, Tehran University of Medical Sciences, Tehran, Iran; 2 Department of Pathology, Shariati Hospital, Tehran University of Medical Sciences, Tehran, Iran

**Keywords:** Biopsy, Carcinoma, Thyroid cancer, Endocrine system diseases, cfDNA, CTCs

## Abstract

Thyroid cancer, one of the most widespread malignancies of the endocrine-related system that over the past three decades, has a vivid increasing rate. The diagnosis and management of it is dependent on the tumor type and stage. Thyroid cancer is divided into four main types, including PTC (papillary thyroid carcinoma), FTC (follicular thyroid carcinoma), MTC (medullarly thyroid carcinoma), and ATC (anaplastic thyroid carcinoma). The development of the noninvasive diagnostic tool for plasma genotyping, also known as “liquid biopsy”, brings a new insight for cancer diagnosis and prognosis. It is mainly containing circulating tumor DNA (ctDNA), circulating tumor cell (CTC), exosomes and extrachromosomal circular DNA (ecDNA). Liquid biopsy as a new plasma genotyping source brings a new prospective of tumor monitoring and therapy. It beneficially reduces the need of tissue biopsy and made early recognition of relapse as well. This article summarizes its components characteristics and their benefit in diagnosis and treatment of thyroid cancer.

## Introduction

**Main components of liquid biopsy**

**Figure1:**
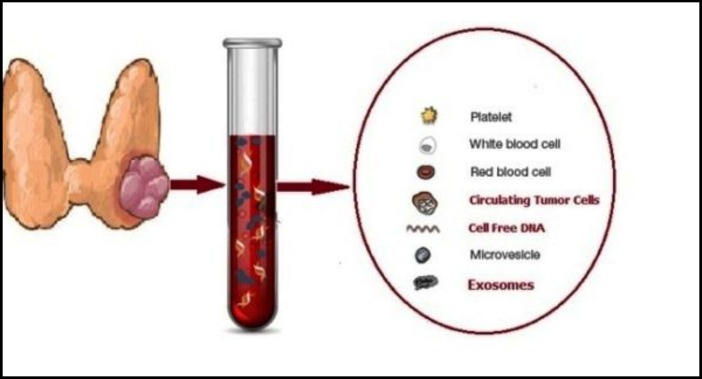
Main components of liquid biopsy for genetic and epigenetic analysis of thyroid tumors.

It is needless to say that tissue biopsies have some weak points like being invasive and useless in understanding metastatic risk, disease progression, and treatment effectiveness more than being hard for repeating ^1^. Over the past few decades, the new real-time diagnostic tool which is referred as ‘‘liquid biopsy’’ has been considered in different type of cancer enormously ^2^^-^^4^. In contrary to analysis of solid tumors requirement as an invasive procedures, blood tests are easy and safe to carry out and several samples can be taken over time. Actually, the concept of liquid biopsy is composed of circulating tumor DNA (ctDNA), circulating tumor cells (CTCs) and exosomes (Figure1) which will be considered in this review in details. 

**Circulating tumor DNA (ctDNA)**

It was in 1940 that for the first time the presence of extracellular or cell-free nucleic acids was recognized by Mandel and Metais ^5^, after that the rheumatologic literature talked about the existence ctDNA in the 1980^6^. Finally, patients with cancer were identified to have high levels of cell-free DNA (cfDNA) in their plasma like patients with benign diseases including inflammatory bowel disease^7^. In fact, ctDNA are calculated to be presented in blood with the length of 160 to 200 base pairs (bp), predominantly 166 bp long that are released by tumor cells into the bloodstream ^8^^-^^10^. They are a genetic representative of tumor which contains the exact genetic defects identical to their original tumor cells. Interestingly, all molecular variations, including point mutations, rearrangements, amplifications and gene copy variations are easily detectable in plasma's ctDNAs. Cell free DNAs are not completely limited to cancer cells for the reasons that live cells naturally shedding DNA fragments as a part of a homeostatic process ^11^^-^^14^ On the other hand, cancer patients usually have far elevated amount of ctDNA than healthy individuals from 0.01% to more than 90%^15^^-^^17^. The logic of this variable amount of ctDNA levels in cancer patients can be connected to the tumor burden of tumor, tumor stage, and efficacy of treatments ^17^^, ^^18^. Although the exact mechanism of coming off ctDNAs into plasma is not clear completely, some suggesting biological processes could be involved, including apoptosis and necrosis from dying cells, or active release from viable tumor cells ^12^^, ^^19^^-^^22^. The cfDNA molecular alterations in plasma can reveal the status of the human body in a timely manner, therefore a study designed to check the background somatic mutations in white blood cells (WBC) and cfDNA for healthy controls ^23^. In order to realize the pattern and source cfDNA mutations, a panel of 50 cancer-associated genes was analyzed in both WBC and cfDNA groups^23^. It was shown that most of mutations in cfDNA originated from WBC and NPM1 gene was the most frequently mutant gene in both WBC and cfDNA^22^^,^^23^.

In normal physiologic conditions, apoptotic and necrotic cells are removed through phagocytes, so ctDNA levels in serum or plasma are quite low, but this mechanism is not applicable in tumor cells. It is possible that in solid tumors ctDNA release through necrosis, autophagy, and other physiologic ^16^^, ^^24^. It should be kept in mind that unlike apoptosis, necrosis DNA fragments are larger because of incomplete and random DNA digestion ^25^. By far, the most interesting spectacle which is brought to the science of oncology by ctDNA is called horizontal tumor gene transfer phenomenon mediated by circulating DNA ^2^^, ^^26^^, ^^27^. It explains that the impact of some other molecules than DNA in tumor formation cannot be ruled out; this is a pure fact that ctDNAs are biologically active DNA to raise tumor progression^26^. In fact, ctDNA represents all genetic alterations which exist in the tumoral genomic DNA, so ctDNA carries genomic and epigenomic alterations such as point mutations, loss of heterozygosity (LOH), rearranged genomic sequences, microsatellite instability (MSI), copy number variation (CNV) and DNA methylation^28^^-^^30^. The results of whole-genome sequencing analysis of ctDNA made it clear that copy number variation (CNV) and Single-nucleotide polymorphisms (SNPs) were noticed in all malignant tumors, but not in healthy individuals^31^. Chan et al. applied shotgun massively parallel sequencing approach in the plasma of cancer patients and successfully completed the whole genome-wide sequencing of CNVs and point mutations^32^. In 1996, recognition of microsatellite instability and loss of heterozygosity in ctDNA were first described by Nawroz et al ^33^. DNA methylation as epigenetic change plays crucial roles in gene expression regulation and genetic alteration^34^^,^^35^. In fact, alteration of DNA methylation in the non-coding and promoter region of genes can be connected with tumor formation, tumor development, and metastatic spread ^36^^, ^^37^. In 1999, unusual DNA methylations were noticed in the plasma and serum of lung ^38^^, ^^39^, breast ^40^ and liver cancers^41^. Afterwards, several researches have pointed out that ctDNA methylation can be considered as an excellent candidate for diagnostic and prognostic of cancer ^29^^,^^42^^-^^44^. Methylation profiling of ctDNA in esophageal cancer patients mentioned the highly significant differences in the methylation status between ctDNA and equivalent tumor tissues^45^. It should be kept in mind that contrary to ctDNA free, RNA molecules are not able to survive in the bloodstream. There is an exception about cell-free microRNAs that can be noticed in plasma or serum of cancer patients^46^. Indeed, detecting RNA molecules could be possible through extracellular vesicles such as exosomes (both coding and non-coding) in platelets^47^^,^^48^. More than cfDNA and cfRNA there are some extrachromosomal circular DNA (ecDNA) which are newly suggested to be presented in blood as a liquid biopsy component ^49^. 

**Circulating Tumor Cells (CTCs)**

The key reason of cancer-associated death is tumor metastasis, unfortunately, the knowledge of this procedure has not completed yet. In fact, dissemination regularly occurs through the blood, so circulating tumor cells (CTCs) as a candidate circulating element are interesting^50^. Circulating tumor cells (CTCs) are circulating cells in the vasculature or lymphatic’s which are released from primary tumors ^51^^, ^^52^. CTCs have the leading role in metastasis which is a key step in the progression of tumors in other distant organs and responsible for the majority of cancer-related deaths^53^. Although for the first time in 1869 CTCs were observed by Thomas Ashworth in the blood of a man with metastatic cancer ^54^, the value of CTCs in modern cancer research instigated in the mid of 1990s by [J. Uhr, UT-Dallas, L. Terstappen and P. Liberti, Immunicon, Philadelphia]. Afterwards, some cancer researches have confirmed that CTCs are derived from primary tumor^55^. Moreover, noteworthy efforts in understanding the biological properties of CTCs have confirmed their critical role in the metastatic spread of carcinoma^56^. Up to now, several technologies with the essential sensitivity and reproducibility to identify CTCs in patients with metastatic disease have recently been developed ^57^^-^^63^. Several studies have shown that the detection of CTCs in the peripheral blood of patients with lung cancer may have prognostic and predicting efficacy in treatment with chemotherapy^39^^, ^^64^^-^^66^.

The ‘seed and soil’ theory which is related to tumor invasion and dissemination was launched in 1889 ^67^. According to this theory, the basic properties of the tumor cells as a seed and host microenvironment as a soil are main determinants of tumor formation sites^56^^,^^68^^,^^69^. Without a doubt, the hypothesis that some CTCs direct ‘tumor-initiating’ process has been supposed because CTCs are proficient to seed detached metastatic disease^70^^, ^^71^. Some reviews of the metastatic process supposed that a reversible epithelial-to-mesenchymal transition (EMT) as a crucial step of metastasis is completely dependent on CTC ^72^^-^^74^. For diagnosis and treatment of breast cancer, CTCs are among the most extensively studied ones^75^^,^^76^. There is a definite correlation between CTCs and breast cancer prognosis and survival^76^. CTCs have been reported to harbor many types of mutations and transformations, but, according to the result of a systematic review, the clinical implication of CTCs molecular characteristics, including Her2, EGFR, CEA, CA15-3, CK19, Ki67, PIK3CA, TGF-β and CXCL1 is more truthful than enumeration of CTCs before and during treatment, especially for making the best personalized treatment decision^76^^-^^79^. Moreover, the change in the number of CTCs in the field of treatment strategies and drug development could be valuable because patients with a remarkable reduction in CTC count after treatment usually show better outcomes^80^^-^^83^.

**Exosomes**

It was established that exosomes are cell-derived nucleic-acid- and protein-rich nanoparticles which are floating in almost all bodily fluids ^84^^, ^^85^. Actually, exosomes are small particles with a diameter of 30 - 100 nm, which is larger than low-density lipoproteins (LDL) and much smaller than red blood cells. The presence of membranous vesicles outside cells in eukaryotic fluids, including blood and urine, was acknowledged 50 years ago although at that time they were assumed as useless products releasing from plasma membrane^86^^, ^^87^. Exosomes can exist in various biological fluids, such as plasma and urine ^88^. At first, exosomes were taken in to account for having role in the removal of needless molecules, after a while some valuable studies clarified exosomes’ complex function in tumor progression and metastasis^88^. They are released from eukaryotic cells when multi-vesicular bodies are fused with the plasma membrane or when they can straightly release from the plasma membrane^89^. The potential of exosomes as a cancer diagnostic tool has been tested for lung cancer^90^ and prostate cancer^91^. Interestingly, the advantage of exosomes is that they are predominant in the bloodstream than CTCs^92^. 

**Liquid biopsy detection and characterizations methods**

The most important step for liquid biopsy analysis is detection and characterization of them in cancer patients. Thanks to recent developments in sequencing technologies like the digital polymerase chain reaction (dPCR) and next-generation sequencing (NGS), now it is easily possible to be detected in blood^93^^-^^95^. Nowadays, numerous dPCR systems which are droplet-based platforms such as QX200 Droplet Digital PCR System (Bio-Rad Laboratories), RainDrop Digital PCR System (RainDance Technologies) with very high sensitivity are industrialized^96^^,^^97^. Moreover, NGS techniques can analyze multiple, broad regions of target ctDNA^94^^,^^98^^,^^99^.Other foremost techniques for detection of mutations in specific genomic regions of ctDNA are “Ion AmpliSeq Technology (Thermo Fisher Scientific)” and “Ion Personal Genome Machine (Ion PGM)”^100^^,^^101^. Also, there are some target capture-based platforms like Sure Select Target Enrichment System (Agilent Technologies) which is generally active for targeted sequencing in combination with the Illumina paired-end sequencing^102^^,^^103^. Interestingly, it was described that Personalized Profiling deep Sequencing of Rearranged Ends can help to the finding of personalized cancer biomarkers ^104^^, ^^105^.

Techniques for detecting Circulating tumor cells are mostly related to the enrichment of CTCs according to different properties of CTCs that discriminate them from other normal hematopoietic cells. Some physical properties are dimensions, density, electric charges, and some biological characteristics are cell surface molecular markers. Epithelial-marker based approaches are the most common practical strategies for CTC detection based on epithelial markers like Cell Search system which is the only FDA-approved platform for CTC detection in clinical practice on patients with breast, prostate, and colorectal cancers^106^. Moreover, presentation of a mixture of different epithelial markers could be helpful to recover additional epithelium-originating tumor cells^107^. Low blood volume as limiting step can also be solved by Cell Collector which used EpCAM antibody-coated wire to capture CTCs in vivo^108^. There is also the chip-based platform CTC-iChip that is an excellent combination of size-based selection and label-dependent enrichment^109^. Additional sized-based approaches are ISET^110^, Screen Cell and Can Patrol^111^, Parsotix^112^ and JETTA ^93^ systems.

Over the past few decades, many techniques have been developed in order to characterization of exosomes from biological fluids. Usually, biophysical methods are zoomed on the exosomal size range like optical particle tracking which is a method that quantify the size of exosomes from 10 nm to 2 µm and the velocity of the particles^113^^-^^119^. Additionally, some microfluidic-based methodologies could be used for exosomal characterization as well^120^^-^^122^. More than exosomal size, the exosome specific molecular markers like proteins and nucleic acids are suitable markers for tumor tracking. As a matter of fact, exosomes are released through both normal and cancerous cells and include several membrane and cytoplasmic proteins. Consequently, its proteins like Enolase 1, Heat shock protein 8 (HSPA8), α (cytosolic), and class A member 1 (HSP90AA1) can be important in clinical diagnostics^123^^, ^^124^. Generally, it could be said that exosomal proteins are allocated to the different functional categories such as tetraspanins (CD9, CD63 and CD81), heat shock proteins (HSC70 and HSC90), membrane transporters (GTPases) and lipid-bound proteins^125^. Not only exosomes are involved in the pathogenesis of cancers but also they are involved in neurodegenerative pathologies, including Alzheimer’s, Parkinson’s and Creutzfeldt-Jakob diseases^126^. Exosomal microRNAs can be useful for diagnostic of several cancer types, for example, some miRNAs were distinguished to be particular biomarkers of ovarian cancer^127^^,^^128^. In patients with lung adenocarcinoma, prostate cancer and esophageal squamous cell cancer (ESCC), the levels of exosomal miRNAs have increased ^129^^-^^131^. Also, exosomal microRNAs may be possible indicative biomarkers for renal fibrosis ^132^ and heart failure^133^. Several companies have improved different technologies for ctDNA, CTCs and detection and characterization of exosomes (Table1).

**Table 1:** The liquid biopsy detection and characterization techniques in experimental applications

**Technique**	**Descriptions**
CTC-Chip	Capture CTCs by using EpCAM- coated microposts under strict manipulation of velocity and shear force
CTC-iChip	The CTC-iChip is composed of two separate microfluidic devices that house three different microfluidic components engineered for inline operation: DLD to remove nucleated cells from whole blood by size-based deflection by using a specially designed array of posts performed in CTC-iChip1, inertial focusing to line up cells to prepare for precise magnetic separation and magnetophoresis for sensitive separation of bead-labeled WBCs and unlabeled CTCs, which are performed in CTC-iChip2. PLTs, platelets
Adna Test	Adna Test has a combination of antibodies that bind with high specificity and affinity to epitopes or antigens on the relevant cancer cells. After magnetic separation, the enriched cells are lysed and purified several time to make the relevant tumor cell information available in the form of mRNA.
EPISPOT( Epithelial Immuno SPOT)	CTCs are enriched by negative depletion and subsequently cultured on a membrane coated with antibodies that capture the secreted proteins. Afterward, the proteins are readily identifiable by immune fluorescence microscopy using fluorochrome-labeled secondary antibodies targeting the protein of interest.
Photoacoustic flowmetry	Making use of the broadband absorption spectrum of melanin, it has been tested to detect melanoma cells and has been combined with nanoparticles targeting cell surface antigens to broaden its applicability in CTC detection.
Affinity based assays Cell Search	The only FDA-approved technology for CTC detection is based on immune magnetic enrichment. It employs an immunomagnetic enrichment step to isolate cells that express the epithelial cells’ adhesion molecule (EpCAM). Additionally, to be identified as a CTC, the cellmust contain a nucleus, express cytoplasmic cytokeratin, and have a diameter larger than 5μm. This technology has demonstrated the prognostic utility of enumerating and monitoringCTC counts in patients with metastatic breast, prostate, and colorectal cancers. Semi-automated analyzer enriches CTCs with ferrofluid nanoparticles coated with anti-EpCAM antibodies, then CD45-, CK8+, CK18+ and CK19+ cells are counted by a four-color semi-automated fluorescence microscope
DEPArray (SiliconBiosystems)	DEPArray™ technology is based on the ability of a non-uniform electric field to exert forces on neutral, polarizable particles, such as cells, that are suspended in a liquid. This electrokinetic principle, called dielectrophoresis (DEP), can be used to trap cells in DEP “cages” by creating an electric field above a subset of electrodes in an array that is in counter phase with the electric field of adjacent electrodes. When a DEP cage is moved by a change in the electric field pattern, the trapped cell moves with it.
MagSweeper	A magnetic stir bar coated with an antibody to EpCAM. The device can process 9 mL of blood per hour and purified cells of interest can be individually selected for subsequent molecular analysis, since the MagSweeper technology preserves cell function and does not perturb gene expression.
Telomescan	A novel cancer detection platform that measures telomerase activity from viable CTCs captured on a parylene-C slot microfilter. Using a constant low pressure delivery system, the new microfilter platform is capable of cell capture from 1 mL of whole blood in less than 5 min, achieving 90% capture efficiency. Addition of an adenovirus-containing GFP to peripheral blood assay, incubation with cancer cells allows precise enumeration and visualization of CTCs.

**Thyroid Cancer**

Thyroid cancer is the most common malignancy of the endocrine system with the remarkable increasing incidence rate over the last three decades^134^^,^^135^. According to the National Cancer Institute, the incidence of thyroid cancer has gotten higher with annually death rate of 0.8% from 2002 to 2011^136^^-^^138^. More often than not, thyroid cancer is diagnosed through Fine Needle Aspiration (FNA) biopsy, and tissue biopsy is classified into four main types, including 70% to 80% of thyroid cancers, papillary thyroid carcinoma (PTC) which is the least aggressive type of cancer^139^^-^^143^, follicular thyroid carcinoma (FTC), which is more aggressive than PTC, medullary thyroid carcinoma (MTC) that develops from C cells in the thyroid gland, and is more aggressive and less differentiated than papillary or follicular cancers and sometimes is associated with multiple endocrine neoplasia 2 (MEN2) and anaplastic thyroid carcinoma (ATC) that is the most dangerous form of thyroid cancer with the high capacity of metastasis to the adjacent lymph nodes and distant sites^140^^,^^144^. Treatment options for thyroid cancer, depending on its type and stage, are surgery, radioactive iodine (131I) therapy, and molecular-targeted therapies with a number of tyrosine kinase inhibitors (TKIs) ^145^. Several genetic and epigenetic alterations could have leading role for thyroid cancer like mutations leading to the activation of the MAPK and PI3K–AKT signaling pathways^146^, MMP2, caspase3^147^^-^^149^, survivin^150^ and nm23^151^. Point mutations of BRAF and RAS genes as well as RET/PTC and PAX8/PPARγ chromosomal rearrangements were found in thyroid cancer ^146^^, ^^152^^-^^154^. In addition to genetic mutations and rearrangements, there are epigenetic modifications which are suggested as important factors for thyroid cancer initiation and progression^149^^, ^^155^.

**Liquid biopsy applications in thyroid cancer management**

In order to real time monitoring of thyroid cancer from diagnosis to post treatment steps, some molecular markers of a noninvasive repeatable biopsy is needed, which means liquid biopsy can be the best candidate. Choosing plasma or serum as a source of cfDNA is challenging because serum apparently contains a greater quantity of free circulating DNA than plasma^156^. The underlying reason for this is unclear, but important because it may have clinical implications in interpreting results and using the appropriate resource^156^. Actually, high levels of circulating cell-free DNA (cf-DNA) have been established to associate with cancer diagnosis and progression. In 2013, it was shown by Mariangela Zane that hypermethylation of SLC5A8 and SLC26A4 genes that are both involved in the iodine metabolism and BRAF^V600E^ mutation in ctDNA have valuable diagnostic value in thyroid cancer patients ^149^^, ^^157^.

Serum DNA methylation assessment as a novel diagnostic tool for thyroid cancer was introduced in 2006^158^. In that research, the evaluation of methylation status of five genes (CALCA, CDH1, TIMP3, DAPK, and RARβ2) been done by real-time quantitative methylation-specific PCR. Finally, they have confirmed the potential efficacy of serum DNA methylation markers as an innovative diagnostic marker for both patients with thyroid nodules and thyroid cancer recurrence in earlier treated patients^158^. Afterwards, the detectable free circulating BRAF in patients with PTC was mentioned as a possible determinant of tumor clinical implication^159^. Moreover, it was explained that decreasing levels of *BRAF*^V600^cfDNA were associated with longer tumor treating field ^160^. A higher amount of circulating mutant *BRAF*^V600^ in plasma was reported as a definite related factor with shorter overall survival in patients who were under BRAF/MEK inhibitors treatment^160^. ATC is so aggressive that needs rapid diagnosis and multimodality management. The University of Texas MD Anderson Cancer Center, between August 2015 and April 2016, run a research in which next-generation sequencing was used in twenty-three patients with ATC^161^. Based on those data, both tumor-based and cfDNA analysis usage in the setting of clinical-trial development and application was suggested^161^. Another aggressive thyroid tumor is medullary thyroid carcinoma which is triggered by activating mutations of the RET proto-oncogene receptor (RET^M918T^ mutation) ^162^^, ^^163^. A cohort study was done by Caitlin Evers on 145 plasma samples from 98 patients (45 RET^M918T^ tumor positive, 25 RET^M918T^ tumor negative and 28 unidentified tumor mutation condition) by using Amplification Refractory Mutation System PCR (ARMS) and the Bio-Rad QX200™ Droplet Digital™ PCR system (ddPCR) (Bio-Rad Laboratories, Hercules, CA). Both ARMS and ddPCR are recommended for plasma DNA analysis in the way of mutation detection during disease progression^162^. For thyroid cancer, personalized medicine approach, interestingly the result of a research had revealed that Vemurafenib have its anti-tumor activity in patients with progressive, circulating BRAF^V600E^ mutation positive refractory to radioactive iodine that had not been treated with a multi-kinase inhibitor drugs ^164^.

Not only circulating DNAs can be valuable source for real-time thyroid tumor tracking but also circulating RNAs have this potential. So, there are some studies which are focused on circulating RNAs in plasma of cancer patients. For example, BRAF^V600E^ as an ordinary mutation of PTC is associated with insistent features of disease^165^. For evaluation of the viability and accuracy of a novel RNA-based blood assay to discriminate individuals with a high-risk tumor mutation in patients with PTC, circulating BRAF^V600E^levels were compared with surgical pathologic DNA-based tissue BRAF mutation assays ^165^. The correlation of the RNA-based blood assay and tissue BRAF status was reported, so this RNA-based blood assay was described as an excellent biomarker for prognosis, surveillance, clinical decision making compared to BRAF-targeted therapies^165^. Additionally, exploring the plasma Long Non-Coding RNA (lncRNAs) for the finding of non-^131^I-avid lung metastases of PTC has been done ^166^. It was shown that two lncRNAs (ENST00000462717 and ENST00000415582) were up regulated and two (TCONS_00024700 and NR_028494) were down regulated in the non-^131^I-avid lung metastases of PTC^166^. 

An interesting case report had illustrated that circulating epithelial cells (CECs) enumeration simplifies the identification and follow-up of a patient with early stage PTCs^167^. A panel of CEC quantification with serum thyroglobulin testing could be a valuable diagnostic marker for monitoring of thyroid cancer patients^167^. Some data make it evident that collective analysis of serum thyroglobulin with CECs, which are EpCAM positive, is completely applicable for patients at disease-free status and the patients with distant metastasis distinguishing ^168^. Therefore, CEC testing thereby can supplement the current standard methods for monitoring disease status of PTC^167^. High-resolution imaging for the detection and characterization of CTCs was used in patients with esophageal, hepatocellular, thyroid and ovarian cancers by Barry M. Dent in January 2016, which resulted in more numbers of CTC detection in the blood of the cancer patient with known metastatic disease^169^. In detail, CTCs were detected in 3 of 6 thyroid cancer patients and most of these tumor cells expressed cytokeratin, thyroglobulin and Sodium: Iodide Symporter (NIS) ^169^. The presence of more than or equal to five CTCs per 7.5 ml of blood in patients with metastatic modularlyTC (metMTC) is associated with inferior overall survival ^170^. Additional research had shown that in metastatic PTC patients CTCs were characterized by aneuploidy, with higher levels of CTCs in metastatic PTC in comparison with controls^171^. Interestingly, the designed probes of lung cancer were suitable for detecting genetic aberrations in metastatic PTC patients’ CTCs that logically could explain the similar lineage-specific chromosomal changes in thyroid and lung malignant progenitor cells^170^. 

Exosomes, 30–120 nm endocytic membrane-derived vesicles, are important for inter-and intra -cellular communication as well as protein and RNA delivery. Because of their role, they have a variety of proteins, nucleic acids, and lipids^172^^, ^^173^. It has been proved frequently that molecular components of exosomes, including exosomal proteins and microRNAs (miRNAs) could be suitable non-invasive biomarkers for clinical diagnosis of tumors ^174^^-^^179^. Very recently, a study revealed that PTC is connected with specific changes in exosomal miRNA profiles^180^. Actually, miRNA-31 was found to be over-represented in the plasma exosomes of PTC compared to benign tumors, while miRNA-21 was helpful for FTC benign tumors discrimination^180^. MiRNA-21 and miRNA-181a-5p were both expressed equally in the exosomes of patients with PTC and FTC; therefore, their assessment will be beneficial to decide between PTC and FTC with 100 % sensitivity and 77 % specificity^180^. Moreover, tumor levels of miR-222 and miR-146b were coupled to the PTC recurrence, whereas miR-222 and miR-146b levels in the circulation were linked to the presence of PTC^181^. Some studies were evidence for exosomes and their cancer-derived miRNAs, which regulated the proliferation of recipient cells. For example, PTC-derived exosomes contain miR-146b and miR-222, which alter proliferation of other cells in a malignant behavior ^182^.

## Conclusion

 Taking everything into consideration now is the exact time to be focused on liquid biopsy for thyroid cancer management. It is really important that liquid biopsy will improve the thyroid cancer diagnostic and prognostic strategies in the minimally non-invasive way.
